# Non-neutral processes drive the nucleotide composition of non-coding sequences in *Drosophila*

**DOI:** 10.1098/rsbl.2008.0174

**Published:** 2008-05-27

**Authors:** Penelope R Haddrill, Brian Charlesworth

**Affiliations:** Institute of Evolutionary Biology, School of Biological Sciences, University of Edinburgh, Ashworth LaboratoriesKing's Buildings, Edinburgh EH9 3JT, UK

**Keywords:** GC content, biased gene conversion, selection, *Drosophila*

## Abstract

The nature of the forces affecting base composition is a key question in genome evolution. There is uncertainty as to whether differences in the GC contents of non-coding sequences reflect differences in mutational bias, or in the intensity of selection or biased gene conversion. We have used a polymorphism dataset for non-coding sequences on the X chromosome of *Drosophila simulans* to examine this question. The proportion of GC→AT versus AT→GC polymorphic mutations in a locus is correlated with its GC content. This implies the action of forces that favour GC over AT base pairs, which are apparently strongest in GC-rich sequences.

## 1. Introduction

A basic feature of an organism's genome is its nucleotide base composition, usually measured by the fraction of base pairs that are GC versus AT. This is highly variable among different parts of eukaryotic genomes. In particular, it tends to be reduced in regions of the genome with low levels of recombination, such as those around the centromeres (Díaz-Castillo & Golic 2007). Two main hypotheses have been proposed to explain such variation in GC content. The first involves differences in patterns of mutational bias. For selectively neutral sequences, the expected fraction of GC versus AT in a given region of the genome is determined by the ratio of the mutation rate for GC→AT to that for AT→GC; this ratio is the mutational bias parameter *κ*, (Sueoka 1962; Li 1987; Bulmer 1991). Differences in GC content can be caused by differences in *κ*, which can be estimated from patterns of nucleotide substitutions (Singh *et al*. 2005), and is generally larger than 1.

Alternatively, GC may be favoured over AT, owing to either natural selection, as with synonymous coding sequence sites (Akashi 1995), or biased gene conversion (BGC). BGC occurs when heterozygotes for GC and AT variants at a nucleotide site produce more than 50% of the GC variant in their gametes, as a result of biased repair of DNA heteroduplexes (Marais 2003). BGC causes an expected change in the frequency of GC versus AT variants at a site similar to that caused by selection (Gutz & Leslie 1976). The greater the intensity of selection or BGC in favour of GC, compared with mutation and genetic drift, the higher the equilibrium GC content of a sequence (Li 1987; Bulmer 1991).

Data on both interspecies divergence and within-species polymorphism permit the detection of selection/BGC, since these forces are less effective at preventing disfavoured variants (AT in this case) entering the population as polymorphic variants than at preventing them becoming fixed (Akashi 1995). If these forces are acting, we should therefore see more GC→AT relative to AT→GC variants among polymorphisms, compared with substitutions between species. Equilibrium for base composition implies an equal number of GC→AT and AT→GC substitutions along a lineage, regardless of the action of selection or BGC. An excess of GC→AT over AT→GC for polymorphisms then indicates the action of selection/BGC (Akashi 1995).

This allows the estimation of the intensity of selection or BGC per site (multiplied by four times the effective population size, *N*_e_) from the proportion of GC→AT polymorphisms among GC→AT and AT→GC polymorphisms (Maside *et al*. 2004). This scaled estimate of selection/BGC is denoted by *γ*. Other methods for estimating *γ* use information on the frequency distribution of variants in the population (Akashi 1999; Galtier *et al*. 2006). A difficulty is that the assumption of equilibrium is often violated; this is known to be the case, for example, for both *Drosophila melanogaster* (Akashi *et al*. 2006) and humans (Duret *et al*. 2006).

Here, we present an analysis of a dataset on polymorphisms in non-coding sequences in a sample of *Drosophila simulans* from Madagascar, together with estimates of divergence from their homologues in *Drosophila melanogaster* and *Drosophila yakuba*. We detect the signature of selection/BGC, especially for sequences with high GC content.

## 2. Material and methods

### (a) Source of data

We used a total of 44 X-linked non-coding loci from the dataset of Haddrill *et al*. (in press), including 23 introns, ten 5′ untranslated transcribed regions (UTRs) and eleven 3′UTRs. Each locus was surveyed in a sample of 20 *D. simulans* males from the putatively ancestral Madagascan population (Dean & Ballard 2004) and was aligned with the homologous *D. melanogaster* (http://flybase.org/, release 4.2) and *D. yakuba* (http://insects.eugenes.org/species/blast) sequences, as described in Haddrill *et al*. (in press).

### (b) Data analysis

To polarize the origin of polymorphisms within *D. simulans*, and to determine the fixed differences between *D. simulans* and *D. melanogaster* that occurred along the *D. simulans* lineage, we reconstructed a *D. melanogaster*–*D. simulans* ancestral sequence using *D. yakuba* as an out-group and estimated the number of polymorphisms and substitutions, as described in Haddrill *et al*. (in press). This allows us to classify both polymorphisms and substitutions along the *D. simulans* lineage as GC→GC, AT→AT, GC→AT or AT→GC. Only the latter two classes are of interest for our purposes. The results for each locus are presented in the electronic supplementary material 1. We estimated *γ* using the maximum-likelihood method of Maside *et al*. (2004).

## 3. Results

We tested for base composition equilibrium by examining the pattern of GC→AT versus AT→GC substitutions along the branch of the phylogeny leading to *D. simulans* from its common ancestor with *D. melanogaster* (see §2). Table 1 shows the numbers of different types of substitutions inferred for the three classes of non-coding DNA. Introns and 5′UTR sequences show no significant departure from the 1 : 1 ratio of GC→AT versus AT→GC substitutions expected under equilibrium base composition; there is a marginally significant (*Χ*^2^=4.24, *p*<0.05) deficit of GC→AT substitutions for 3′UTR sequences. For the pooled dataset, there is no significant departure from equality (*Χ*^2^=2.21), and there is no significant heterogeneity among the three classes of sequence. If we divide the set of loci into three nearly equal-sized classes with respect to their GC contents, then there are no significant differences among the high, medium and low GC content loci. Overall, there is no evidence for an excess of GC→AT over AT→GC substitutions, consistent with Akashi *et al*. (2006).

We then asked whether there is an excess of GC→AT over AT→GC mutations among polymorphisms. If base composition is close to equilibrium, this is an indicator of selection or BGC in favour of GC base pairs (see §1). Table 1 shows the numbers of polymorphic variants in the three different classes of non-coding sequence. For introns and 3′UTR sequences there is no significant departure from 1 : 1 (*Χ*^2^=2.33 and 0.29, respectively), whereas for 5′UTR sequences, *Χ*^2^=4.97 (*p*<0.02; all *p* values for tests of 1 : 1 ratios are one tailed). If all sequences are pooled, *Χ*^2^=5.86 (*p*<0.01); this result is not simply due to the 5′UTR sequences alone, since we find no significant heterogeneity between the 5′UTR sequences and the introns and 3′UTR sequences combined (*Χ*^2^=1.72, *p*>0.05). This suggests that GC is favoured over AT.

The three classes of non-coding sequences differ in their GC content, with means of 37, 40 and 50% for intronic, 3′UTR and 5′UTR sequences, respectively. If we pool the three classes, and divide the data into high, medium and low GC content sequences as above, the *Χ*^2^ values for 1 : 1 for polymorphisms in each category are 9.48, 1.33 and 0.09, respectively. The first of these has *p*<0.001. The heterogeneity *Χ*^2^ between these categories is 4.84 (*p*<0.05). The proportion of GC→AT among GC→AT and AT→GC polymorphisms at a locus is significantly correlated with its GC content (figure 1*a*).

This suggests that there is a tendency for GC-rich sequences to be associated with stronger selection/BGC in favour of GC. We investigated this by estimating the scaled selection parameter *γ* (see §2). We first fitted a common *γ* to all the loci, obtaining a maximum-likelihood estimate of *γ*=0.25, ln *L*=−496.83, with two-unit support limits 0.04–0.47. Table 2 shows the results of fitting separate *γ* values to the three categories of GC content, with strong support for a positive *γ* only for the high GC content sequences (the lower three-unit support limit in this case is 0.13). The difference in fit between the models with a common *γ* and with individual values fitted is significant (*Χ*_2_^2^=5.04, *p*<0.02 on a one-tailed test).

If GC-rich sequences are associated with stronger selection/BGC in favour of GC, we would expect to see GC variants present at a higher mean frequency in this category, compared with the medium and low GC content categories (Galtier *et al*. 2006). For each category, we summed the number of variants in the GC and AT states across every GC/AT polymorphic site, and compared the results with the neutral expectation of equal numbers. All three categories show an excess of variants in the GC state compared with the AT state (GC contents: high, *Χ*^2^=173.25; medium, *Χ*^2^=33.93; low, *Χ*^2^=31.93; all *p*<0.001), and a test of heterogeneity indicates that this deviation differs between categories (*Χ*^2^=49.36, *p*<0.001). We also find a positive correlation between the mean frequency of GC variants for a locus and its GC content (figure 1*b*), as expected if GC-rich sequences are associated with stronger selection/BCG in favour of GC. Given the potential biases associated with ancestral inference (Eyre-Walker 1998; Akashi *et al*. 2007), we can also use these unpolarized data to recalculate *γ*, using the method of Cutter & Charlesworth (2006). Estimates are not significantly different from those reported above (see the electronic supplementary material 2), indicating that inference biases do not account for the patterns that we see.

## 4. Discussion

Our results suggest that non-coding sequences with high GC contents are associated with stronger selection/BGC in favour of GC than sequences with low or intermediate GC contents. It is of interest to compare the observed GC contents with the equilibrium values predicted from the Li–Bulmer equation, 1/(1+*κ* exp−*γ*) (Li 1987; Bulmer 1991). The results of Singh *et al*. (2005) suggest an estimate of *κ* of 2.1 for non-functional sequences in heterochromatic regions with very low GC content in *D. melanogaster*; these are the least likely to be affected by gene conversion, although it may not be completely absent (Gay *et al*. 2007). If we use this value of *κ* in conjunction with the maximum-likelihood estimates of *γ* in table 2, the predicted values of GC contents are 33, 37 and 46%, for the low, medium and high GC sequences, respectively. These agree with the mean GC contents for these regions (table 1). Use of the standard formula for fixation probabilities of mutations affected by selection (Kimura 1983, p. 43) shows that there is approximately only a 5% underestimation of *κ* from substitution patterns, if we use the maximum-likelihood estimate of *γ* for low GC content from table 2. A *κ* of 2.1 thus seems to fit the data well, and our estimates of *γ* are consistent with the hypothesis that differences in GC content among non-coding sequences reflect differences in the intensity of selection or BGC in favour of GC. Selection in favour of GC would imply functionality of GC base pairs in non-coding sequences, but there is currently no way to distinguish between selection and BGC using these data.

These results contrast with those of Galtier *et al*. (2006) who used a different method to analyse a *D. melanogaster* non-coding polymorphism dataset for an African population (Glinka *et al*. 2003). Their best-fitting model gave *γ* and *κ* estimates of between 1.5 and 1.7 and between 3 and 3.7, respectively, for low, medium and high GC content sequences. These predict a GC content of 59%, very different from the observed values. The most likely explanation of these discrepancies is that this population is not at statistical equilibrium, for which there is other evidence (Li & Stephan 2005). Indeed, it is unlikely that *γ* for non-coding sequences could be as high as the estimates of Galtier *et al*. (2006), since values for synonymous sites are of the order of 2 in several *Drosophila* species (Maside *et al*. 2004; Bartolomé *et al*. 2005; Comeron & Guthrie 2005), and these include the effects of both BGC and selection on codon usage bias. The same reservation applies to the estimates of *γ* obtained for humans by Lercher *et al*. (2002).

## Figures and Tables

**Figure 1:**
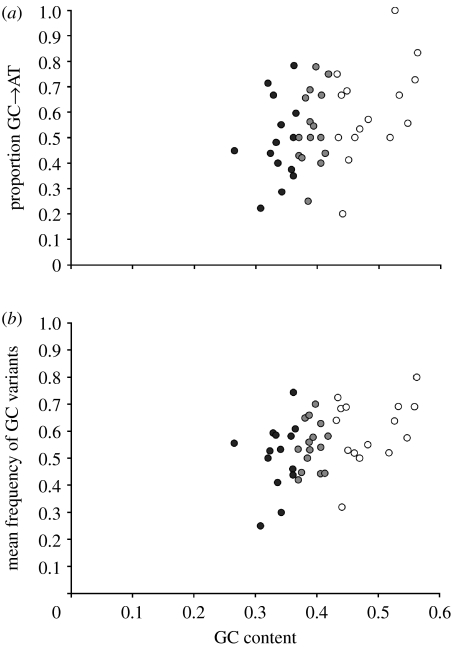
Relationships between the GC content of a locus and (*a*) the proportion of GC→AT among GC→AT and AT→GC polymorphisms and (*b*) the mean frequency of GC variants. The Spearman's rank correlations are 0.377 (*p*<0.01) and 0.355 (*p*<0.01), respectively. Black circles, low GC; grey circles, medium GC; white circles, high GC.

**Table 1:** Numbers of GC→AT and AT→GC substitutions and polymorphisms in different classes of sequences. (High, medium and low GC content sequences correspond to loci with GC contents in the ranges 43–56% (*n*=15, mean 45%), 37–42% (*n*=15, mean 39%) and 26–36% (*n*=14, mean 34%), respectively.)

class of sequence	substitutions GC→AT	substitutions AT→GC	polymorphisms GC→AT	polymorphisms AT→GC
intron	37	44	265	231
5′UTR	24	24	81	55
3′UTR	11	23	47	42
*GC content*				
high	19	22	119	76
medium	32	38	136	119
low	21	31	138	133

**Table 2:** Maximum-likelihood estimates of the scaled selection parameter *γ*

GC content category	maximum likelihood *γ*	maximum log-likelihood	two-unit support limits
high	0.60	−130.38	(0.21–1.00)
medium	0.20	−176.19	(−0.16–0.54)
low	0.05	−187.80	(−0.35–0.38)

## Supplementary Material

Additional data Polymorphism, substitution and GC content data per locus

Additional table Estimates of the scaled selection parameter gamma, calculated using the method of Cutter & Charlesworth 2006
