# Supplementary material for: Non-neutral processes drive the nucleotide composition of non-coding sequences in Drosophila
Source: Biol Lett. 2008 May 27;4(4):438–41. doi: 10.1098/rsbl.2008.0174 (PMC2515589; doi:10.1098/rsbl.2008.0174)
Supplement: Supplementary file 1 [file rsbl20080174s63.doc]

**Electronic Supplementary Material 2**

Estimates of the scaled selection parameter γ calculated using the method of Cutter and Charlesworth 2006.

| GC content category | maximum likelihood γ | maximum  log-likelihood | 2-unit support  limits |
| --- | --- | --- | --- |
|  |  |  |  |
| High | 0.85 | -438.87 | (0.45 – 1.35) |
| Medium | 0.40 | -569.54 | (0.07 – 0.70) |
| Low | 0.40 | -622.64 | (0.07 – 0.70) |
